# Probing the Optical Properties of MoS_2_ on SiO_2_/Si and Sapphire Substrates

**DOI:** 10.3390/nano9050740

**Published:** 2019-05-14

**Authors:** Tao Han, Hongxia Liu, Shulong Wang, Shupeng Chen, Wei Li, Xiaoli Yang, Ming Cai, Kun Yang

**Affiliations:** 1 Key Laboratory for Wide-Band Gap Semiconductor Materials and Devices of Education, The School of Microelectronics, Xidian University, Xi’an 710071, China; 15639119745@163.com (T.H.); chenshupeng999@126.com (S.C.); li20101467@163.com (W.L.); cm9999787@163.com (M.C.); kuny2019@163.com (K.Y.); 2 The School of Mathematics and Statistics, Xidian University, Xi’an 710071, China; xiaoyangxiaoli163@163.com

**Keywords:** MoS_2_, optical properties, Raman spectrum, PL spectrum, AFM

## Abstract

As an important supplementary material to graphene in the optoelectronics field, molybdenum disulfide (MoS_2_) has attracted attention from researchers due to its good light absorption capacity and adjustable bandgap. In this paper, MoS_2_ layers are respectively grown on SiO_2_/Si and sapphire substrates by atmospheric pressure chemical vapor deposition (APCVD). Atomic force microscopy, optical microscopy, and Raman and photoluminescence spectroscopy are used to probe the optical properties of MoS_2_ on SiO_2_/Si and sapphire substrates systematically. The peak shift between the characteristic A_1g_ and E^1^_2g_ peaks increases, and the I peak of the PL spectrum on the SiO_2_/Si substrate redshifts slightly when the layer numbers were increased, which can help in obtaining the layer number and peak position of MoS_2_. Moreover, the difference from monolayer MoS_2_ on the SiO_2_/Si substrate is that the B peak of the PL spectrum has a blueshift of 56 meV and the characteristic E^1^_2g_ peak of the Raman spectrum has no blueshift. The 1- and 2-layer MoS_2_ on a sapphire substrate had a higher PL peak intensity than that of the SiO_2_/Si substrate. When the laser wavelength is transformed from 532 to 633 nm, the position of I exciton peak has a blueshift of 16 meV, and the PL intensity of monolayer MoS_2_ on the SiO_2_/Si substrate increases. The optical properties of MoS_2_ can be obtained, which is helpful for the fabrication of optoelectronic devices.

## 1. Introduction

Graphene has been researched extensively due to its many excellent properties, making it one of the most promising two-dimensional materials [1,2,3]. However, the Dirac energy states at the K point of the graphene Benelux region are in contact with each other, which limit the application of graphene in optoelectronics [4,5]. To compensate for the lack of band gap, researchers have focused their attention on the transition metal dichalcogenides (TMDs). Molybdenum disulfide (MoS_2_) has similar physical properties to graphene, but it contains some advantages that graphene cannot match [6,7,8,9]. The monolayer MoS_2_ is a “sandwich” structure, whereby the upper and lower layers are the hexagonal planes composed of sulfur atoms, and the middle is a layer of metal molybdenum atoms. Each molybdenum atom in the layer bonds with six sulfur atoms through covalent bonding to form the Mitsubishi columnar coordination structure. Meanwhile, each sulfur atom combines with three molybdenum atoms through covalent bonding. The multilayer or block MoS_2_ with an interlayer distance of 0.65 nm is made up of monolayer MoS_2_, which is combined by the weak van der Waals force [10,11]. The band gap of MoS_2_ increases with its thickness decreases, especially when it is reduced to the monolayer, and the band gap changes from the indirect band gap to the direct band gap [12]. In addition, monolayer MoS_2_ has a high electron mobility and luminous efficiency, so it can be used in photovoltaic fields, such as in field effect transistors and sensors [13].

MoS_2_ is a layered semiconductor material with strong light absorption, so it can be applied to photoluminescence, photovoltaic, and photocatalytic research [14]. The layer number and substrate environment would have a great influence on the electronic structure, physical, and optical properties of MoS_2_, which would also affect the performance of the optical device [15]. At present, there are few studies on the optical properties of MoS_2_. Therefore, the use of MoS_2_ on SiO_2_/Si and sapphire substrates under different laser wavelengths and powers has been researched in an attempt to understand the luminescence laws of MoS_2_.

In this paper, different layers of MoS_2_ are grown on SiO_2_/Si and sapphire substrates by atmospheric pressure chemical vapor deposition (APCVD) [16]. The effects of different laser wavelengths, powers, layer number, and substrate on the optical properties of MoS_2_ are researched by the optical microscopy (OM), Raman spectroscopy (Raman), photoluminescence spectroscopy (PL), and atomic force microscopy (AFM), systematically, to master the luminescence laws. First, spectral characteristics of monolayer MoS_2_ on the SiO_2_/Si and sapphire substrates are each studied; then, spectral characteristics of MoS_2_ with different layers on the SiO_2_/Si and sapphire substrates are also tested; subsequently, the spectral properties of MoS_2_ with different layers under different excitation wavelengths are researched; finally, the optical characteristic laws of MoS_2_ are summarized, which can help in the fabrication of optoelectronic devices.

## 2. Experimental Methods

**Figure 1:**
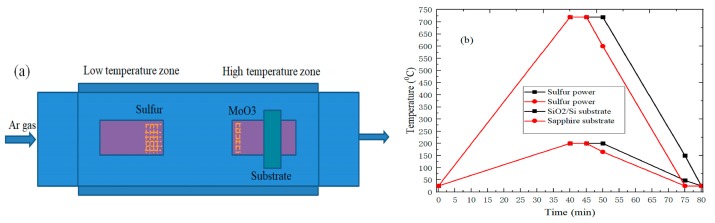
(**a**) The schematic diagram of MoS_2_ growth; (**b**) The change curve of experimental temperature.

The specific experiment processes are as follows: single crystal sapphire substrate (C<0001>, 99.999%, and hexagonal lattice structure, 6Carbon Technology, Shenzhen, China), and silicon substrate with a thickness of 285 nm silicon dioxide (6Carbon Technology, Shenzhen, China) are selected in this experiment. Firstly, the SiO_2_/Si and sapphire substrates were sequentially placed in acetone, deionized water, absolute ethanol, and deionized water for ultrasonic cleaning for 10, 5, 10, and 5 min, respectively, and dried with nitrogen gas gun [17,18]. Then, 100 mg sulfur powder (99.5%, Alfa Aesar, Shanghai, China) and 2 mg molybdenum trioxide powder (MoO_3_, 99.95%, Alfa Aesar, Shanghai, China) were separately weighed using an electronic analytical balance and each placed into two different quartz boats. The quartz boat containing sulfur powder was placed in the upstream low-temperature zone center of the tube furnace. Subsequently, substrate was placed, face-down, at 5 cm downstream from the MoO_3_ powder for, and then the quartz boat with face-down substrate and MoO_3_ powder was placed in the downstream high-temperature zone center of the tube furnace. The specific location of the experimental material is shown in Figure 1a. Afterwards, high-purity argon gas (99.999%) with a flow rate of 200 sccm was introduced into the quartz tube for 10 min to remove the air of the tube furnace [19]. Figure 1b shows the reaction temperature change, and the temperature in the low-temperature zone was set to 200 °C, and the heating rate was 4.38 °C/min. At the same time, the temperature in the high-temperature zone was set to 720 °C, and the heating rate was 17.5 °C/min. The growth time of sapphire and SiO_2_/Si substrate were maintained for 5 and 10 min under the growth temperature of 720 °C, respectively. Finally, the temperature of the tube furnace was cooled to room temperature naturally after the growth reaction was complete.

Different layer numbers of MoS_2_ on SiO_2_/Si and sapphire substrates were systematically characterized by OM, AFM, and Raman and PL spectroscopy [20]. The test characterization experiment of MoS_2_ was the high-resolution Raman spectroscopy of LabRam HR Evolution model (HORIBA Jobin Yvon, Paris, France) using a high-definition color camera, which can achieve all functions of a standard microscope. Under the premise of maintaining constant room temperature, the specific test conditions of Raman spectroscopy are as follows: laser wavelength of 532 and 633 nm; spot diameter of 1 μm; spectral resolution ≤ 0.65 cm^−1^; scan time of 5 s; and the accumulation number of 3 s. In addition, the AFM (Dimension Icon, NanoScope8.10, Beijing, China) with 2% scanning accuracy error was also used to explore the size, thickness, surface morphology, and properties of MoS_2_ grown on sapphire and SiO_2_/Si substrates [21].

## 3. Results and Discussion

### 3.1. Monolayer MoS_2_ on Sapphire Substrate and SiO_2_/Si Substrates

The SiO_2_/Si and sapphire substrates are suitable substrate materials for growing high-quality, large-area, uniform triangles of MoS_2_. In addition, the surface topography of MoS_2_ on the SiO_2_/Si and sapphire substrates can easily be observed by optical microscopy.

**Figure 2:**
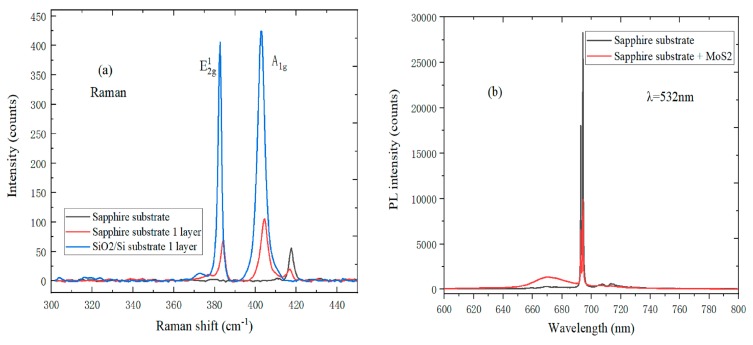
(**a**) Raman spectrum of monolayer MoS_2_ on SiO_2_/Si and sapphire substrates; (**b**) PL spectrum of MoS_2_+sapphire and sapphire substrate at the 532 nm laser wavelength.

Raman spectroscopy can determine the layer number, layer stress, and doping level of MoS_2_, so the Raman spectrum can further help us to master the structural characteristics of MoS_2_. In Figure 2a, the Raman spectrum peak of monolayer MoS_2_ on a sapphire substrate is much weaker than that on the SiO_2_/Si substrate; the reason is that the sapphire substrate is transparent, and the Raman spectrum collects the reflected light from MoS_2_. It can be seen from Figure 2b that the PL spectrum of MoS_2_+sapphire is formed by the combination between monolayer MoS_2_ and sapphire substrate. The B peak corresponds to the direct jump of B exciter due to the valence band splitting at the K point in the Brillouin zone [22]. The B peak of MoS_2_+sapphire PL spectrum is much stronger than that of a pure sapphire substrate, which can be explained by the strong coupling between MoS_2_ and sapphire substrate, and the efficient energy transfer occurs in MoS_2_ samples on the sapphire substrate.

### 3.2. Characterization of Monolayer MoS_2_ on SiO_2_/Si Substrate

**Figure 3:**
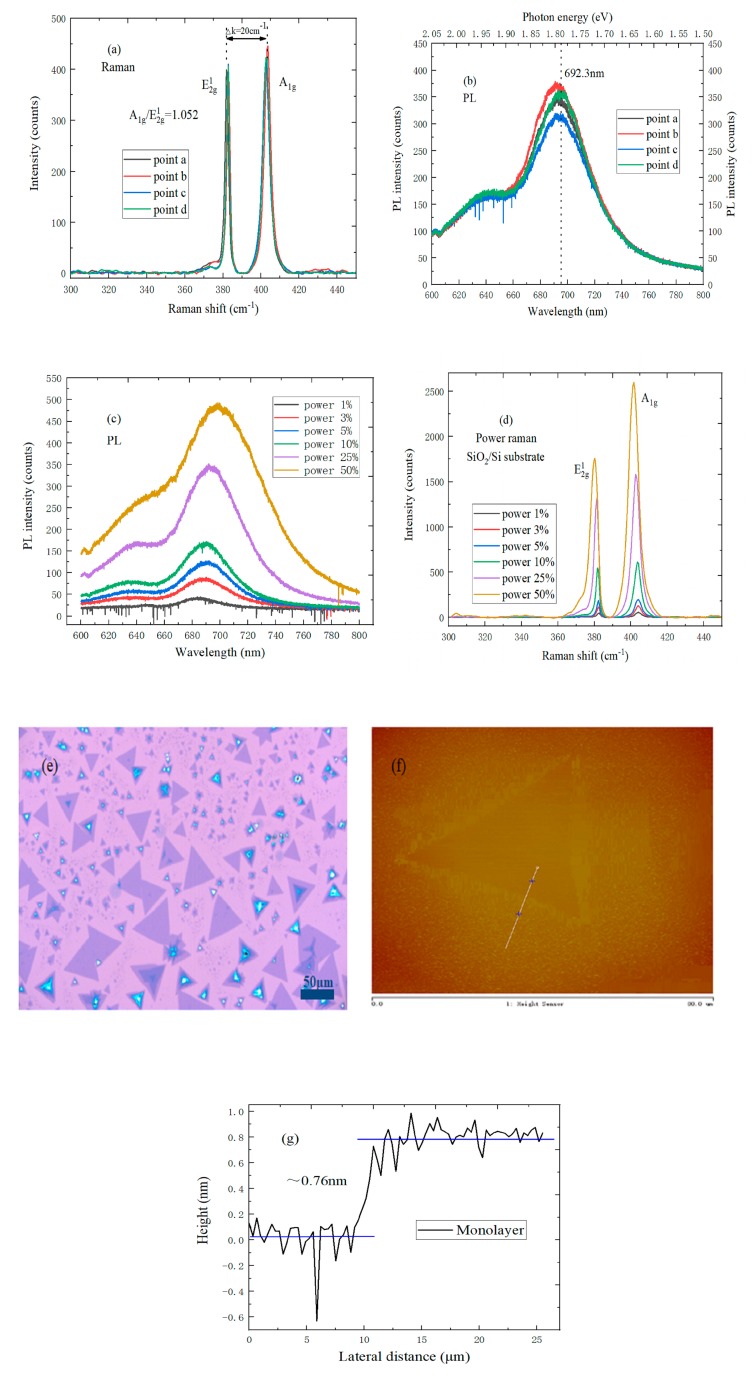
(**a**) Raman spectrum of four different points in monolayer MoS_2_ on a SiO_2_/Si substrate; (**b**) PL spectrum of four different points in monolayer MoS_2_ on a SiO_2_/Si substrate; (**c**) PL spectrum of monolayer MoS_2_ on a SiO_2_/Si substrate with different laser power; (**d**) Raman spectrum of monolayer MoS_2_ on a SiO_2_/Si substrate with different laser power; (**e**) Optical micrograph of monolayer MoS_2_ on a SiO_2_/Si substrate; (**f**) AFM morphology of monolayer MoS_2_ on a SiO_2_/Si substrate; (**g**) Height profile of monolayer MoS_2_ on a SiO_2_/Si substrate.

In Figure 3a, the Gauss–Lorentz function is used to fit the Raman spectrum. There are two characteristic peaks in the Raman spectrum, the E^1^_2g_ and A_1g_ peak, and the peak shift difference between E^1^_2g_ and A_1g_ was 20 cm^−1^, and the ratio A_1g_/E^1^_2g_ = 1.052 ≈ 1, which can help in establishing that the MoS_2_ sample is monolayer.

There exist I and B exciton peaks on a SiO_2_/Si substrate, and the PL spectrum of monolayer MoS_2_ is dominated by free exciton transition luminescence at room temperature.

(1) $$E=h\times H=\frac{h}{k}\times \frac{c}{\lambda }=\frac{1243}{\lambda }$$

The Planck constant, wavelength, energy, constant, and light speed are respectively represented by the symbols *h*, *λ*, *E*, *k*, and *c* in Equation (1), and the units of *h*, *λ*, *E*, *k*, and *c* are J.s, nm, eV, J/eV, and nm/s, respectively. According to the conversion relationship between photon energy and laser wavelength [23], it can be found from Figure 3b that the strongest photoluminescence peak is at 692.3 nm, and the corresponding photon energy is about 1.8 eV. In addition, a B exciton peak exists near 2.0 eV due to energy band splitting, which is the same as the reported band gap of monolayer MoS_2_ [24]. In Figure 3c, the I exciton peak is dominant in PL spectrum intensity under the low excitation power. Meanwhile, the B exciton peak position and I exciton peak shape did not change much when the excitation power increased, but the shape of the B exciton peak changed significantly. Moreover, the relative position between the I exciton peak and B exciton peak was redshifted to some extent when the laser power increases, which can be explained by the introduction of n-type doping MoS_2_ on the SiO_2_/Si substrate. Figure 3d shows the Raman spectrum of monolayer MoS_2_ with different laser power. When the laser power was increased, the Raman spectrum peak intensity increased, and the E^1^_2g_ characteristic peak was blueshifted. The reason is that the MoS_2_ grown on the SiO_2_/Si substrate is an n-type doped semiconductor [25]. Figure 3e shows the uniform triangular monolayer MoS_2_ with a side length of 50 μm on the SiO_2_/Si substrate, which is much larger than monolayer MoS_2_ obtained by the mechanical peeling method [26]. In addition, the surface color of monolayer MoS_2_ is uniform, which is in sharp contrast with the color of the SiO_2_/Si substrate, so it can be determined that the MoS_2_ sample is monolayer. It also can be seen from Figure 3f,g that triangular MoS_2_ has a very uniform color, and the thickness is 0.76 ± 0.015 nm, which can also indicate that the grown MoS_2_ is monolayer.

### 3.3. Characterization of Monolayer MoS_2_ on Sapphire Substrate

**Figure 4:**
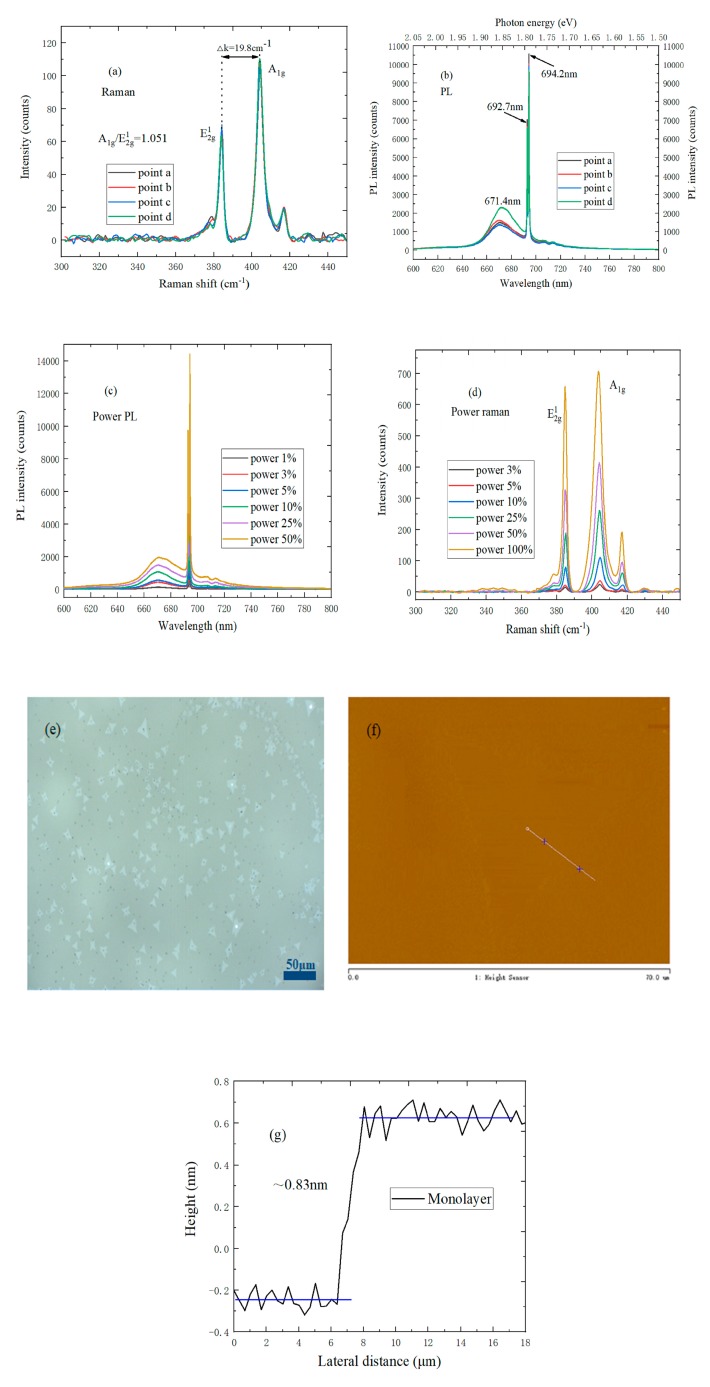
(**a**) Raman spectrum of four different points in monolayer MoS_2_ on a sapphire substrate; (**b**) PL spectrum of four different points in monolayer MoS_2_ on a sapphire substrate; (**c**) PL spectrum of monolayer MoS_2_ on a sapphire substrate at different laser powers; (**d**) Raman spectrum of monolayer MoS_2_ on a sapphire substrate at different laser powers; (**e**) Optical micrograph of monolayer MoS_2_ on a sapphire substrate; (**f**) AFM morphology of monolayer MoS_2_ on a sapphire substrate; (**g**) Height profile of monolayer MoS_2_ on a sapphire substrate.

In Figure 4a, the characteristic Raman peak position difference of monolayer MoS_2_ on a sapphire substrate is 19.8 cm^−1^, and A_1g_/E^1^_2g_ ≈ 1.051, which is basically the same as the Raman spectrum of monolayer MoS_2_ on a SiO_2_/Si substrate. As shown in Figure 4b, the B peak of the PL spectrum on the sapphire substrate has a blueshift of 56 meV compared to the n-type doped monolayer MoS_2_ on the SiO_2_/Si substrate. The reason is that the sapphire substrate does not introduce any doping into the MoS_2_, and the optical transition process was dominated by neutral exciton radiation. It can be found by observing Figure 4c that the photoluminescence peaks at 671 and 693 nm correspond to the B exciton peak and the sapphire substrate, respectively, and the PL intensity of monolayer MoS_2_ is proportional to the laser power, which indicates that the monolayer MoS_2_ on the sapphire substrate did not undergo saturation absorption. This is because there was no charged impurity on the surface of the sapphire substrate, so no doping was introduced into MoS_2_ [27,28]. Figure 4d shows the Raman spectrum of monolayer MoS_2_ on the sapphire substrate at different laser powers. The Raman spectrum peak intensity increases with laser power increases. Unlike the SiO_2_/Si substrate, the characteristic E^1^_2g_ peak does not exist in the blueshift since the sapphire substrate does not introduce any doping into the monolayer MoS_2_. In Figure 4e, the shape of monolayer MoS_2_ is triangular, and the size can be up to 30 μm. As shown in Figure 4f,g, the triangular MoS_2_ has uniform color, and the thickness of MoS_2_ is about 0.83 ± 0.017 nm, which indicates that the MoS_2_ sample is monolayer.

### 3.4. Characterization of Different Layers of MoS_2_ on SiO_2_/Si Substrate

**Figure 5:**
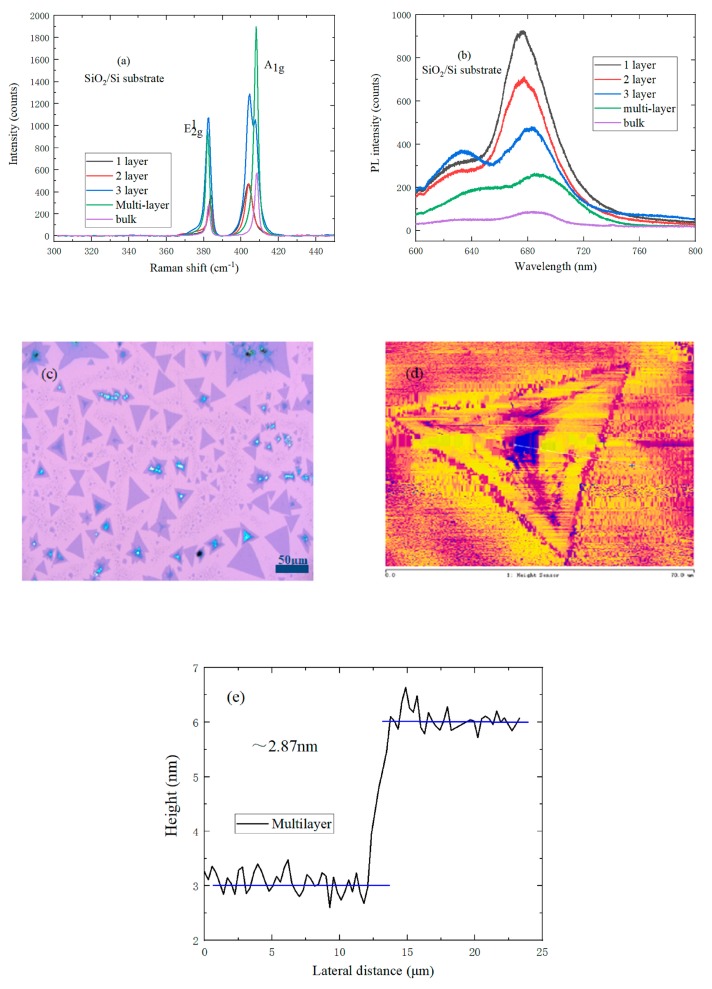
(**a**) Raman spectrum of MoS_2_ with different layers on a SiO_2_/Si substrate; (**b**) PL spectrum of MoS_2_ with different layers on a SiO_2_/Si substrate; (**c**) Optical micrograph of MoS_2_ with different layers on a SiO_2_/Si substrate; (**d**) AFM morphology of multilayer MoS_2_ on a SiO_2_/Si substrate; (**e**) Height profile of multilayer MoS_2_ on a SiO_2_/Si substrate.

In order to analyze the lattice vibration modes, the Raman and PL spectrums of different layer MoS_2_ on the SiO_2_/Si and sapphire substrates were tested. In Figure 5a, the greater the layer number of MoS_2_, the brighter the color under the microscope, which indicates the presence of MoS_2_ with different layer numbers on the surface of the SiO_2_/Si substrate. From bulk to monolayer samples, MoS_2_ had two distinct characteristic peaks in the range of 300~450 cm^−1^: E^1^_2g_ and A_1g_ peaks. The E^1^_2g_ peak corresponds to the vibration of Mo atom and S atom parallel to the layer, and the A_1g_ peak corresponds to the vibration of S atom perpendicular to the layer. The E^1^_2g_ peak with in-plane vibration mode is redshifted when the number of layers of MoS_2_ increase. This is because the short-range van der Waals force is stronger than dielectric shielding of the long-range Coulomb interaction. Meanwhile, the A_1g_ peak of out-of-plane vibration mode has blueshifted with the gradual decrease of van der Waals force [29]. It can be seen from Figure 5b that the photoluminescence of the 2-layer and 3-layer MoS_2_ can also be observed due to the interaction between the layers. The B peak and I peak of monolayer MoS_2_ are respectively located at 627 and 677 nm. The I peak of the PL spectrum corresponds to direct exciton transition at the K point of Brillouin zone, and the B peak corresponds to the B exciton peak direct transition, which is caused by the valence band splitting. The I peak exists and is slightly redshifted when the MoS_2_ layer number increases; this is because the electron band gap at the K point of the Brillouin zone decreases, and the increase in interlayer van der Waals forces. In addition, the PL intensity increases gradually when the MoS_2_ layer number decreases. Therefore, monolayer MoS_2_ has the highest PL quantum yield, which is related to the phonon-dependent carrier band relaxation [30].

(2) $$\eta_{Lum}\approx K_{rad}/\left(K_{rad}+K_{defect}+K_{relax}\right)$$

The $K_{rad}$, $K_{defect}$, and $K_{relax}$ in Formula (2) refer to the radiation recombination rate, defect trapping rate, and carrier relaxation rate, respectively. The $K_{relax}$ decreases as the indirect band gap width increases, which leads to an increase in the luminescence intensity and an essential change in the electronic structure of MoS_2_. Therefore, the monolayer MoS_2_ is $K_{relax}=0$. Figure 5c shows the optical micrograph of MoS_2_ with different layer numbers on the SiO_2_/Si substrate. The triangular shape of MoS_2_ has a distinct color contrast with the SiO_2_/Si substrate, and the size can be up to 30 μm. As shown in Figure 5d,e, the color of triangular MoS_2_ is not uniform, and the thickness of middle MoS_2_ is about 2.87 ± 0.057 nm, which indicates that the grown MoS_2_ is a multilayer.

### 3.5. Characterization of Different Layers MoS_2_ on Sapphire Substrate

**Figure 6:**
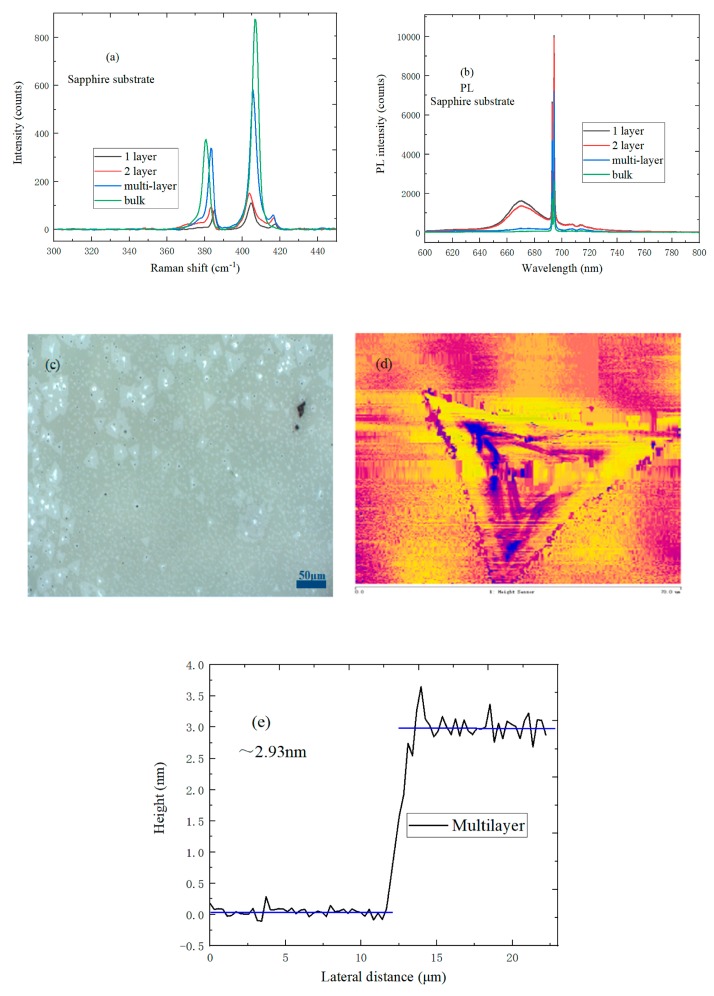
(**a**) Raman spectrum of MoS_2_ with different layers on a sapphire substrate; (**b**) PL spectrum of MoS_2_ with different layers on a sapphire substrate; (**c**) Optical micrograph of MoS_2_ with different layers on a sapphire substrate; (**d**) AFM morphology of multilayer MoS_2_ on a sapphire substrate; (**e**) Height profile of multilayer MoS_2_ on a sapphire substrate.

In Figure 6a, the interlayer van der Waals force increase gradually with the increase of MoS_2_ layer number, which would result in an increase of the mechanical constant and redshifting of the A_1g_ peak. Meanwhile, the the characteristic E^1^_2g_ peak redshifts to some extent; this is because long-range Coulomb interactions play a more prominent role in the control of the atomic vibration when the MoS_2_ layer numbers increase. Therefore, the frequency shift difference between the characteristic E^1^_2g_ and A_1g_ peaks on the sapphire substrate increase monotonically with the increase of MoS_2_ layer number. It is not difficult to find, when observing Figure 6b, that the bulk of the MoS_2_ on the sapphire substrate does not exist in the PL peak. When the MoS_2_ layer number decreases, MoS_2_ change from an indirect band gap to direct band gap, and the PL intensity increases gradually, so the PL intensity of monolayer MoS_2_ can be up to its maximum value. The B peak of monolayer MoS_2_ exists in the blueshift region, slightly, due to the annihilation of the phonon-assisted decay channel. The 1- and 2-layer MoS_2_ on the sapphire substrate has a higher PL peak intensity than that of 1- and 2-layer MoS_2_ on the SiO_2_/Si substrate. The reason is that the relative dielectric constant of the sapphire substrate is higher than that of the SiO_2_/Si substrate, which would result in the higher dielectric shielding effect. Figure 6c shows the optical micrograph of MoS_2_ with different layers on the sapphire substrate, where the size of MoS_2_ triangles are mostly 20 μm, and the surface of MoS_2_ sample is relatively clean and uniform, which is in sharp contrast with the sapphire substrate. As shown in Figure 6d,e, the color and thicknesses of triangular MoS_2_ are not uniform, and the middle thickness of MoS_2_ is about 2.93 ± 0.059 nm, which indicates that the MoS_2_ sample is multilayered.

### 3.6. Characterization of MoS_2_ with Different Layers on Sapphire Substrate Under the 633 nm Laser Wavelength

**Figure 7:**
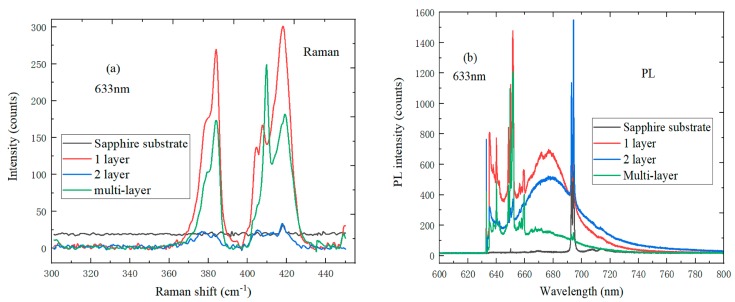
(**a**) Raman spectrum of MoS_2_ with different layers on the sapphire substrate; (**b**) PL spectrum of MoS_2_ with different layer on the sapphire substrate under the laser wavelength of 633 nm.

In Figure 7a, the Raman spectrum of MoS_2_ with different layers on the sapphire substrate under the 633 nm laser wavelength, and the peak position of the MoS_2_ Raman spectrum are closely related to its thickness. Due to the increase in van der Waals forces, the characteristic E^1^_2g_ peak blueshifted while the characteristic A_1g_ peak redshifted when the MoS_2_ layer number increased. It was possible to determine the layer number of MoS_2_ according to the wavenumber difference of A_1g_ − E^1^_2g_. Due to the very small local field effect, monolayer MoS_2_ had the strongest PL intensity. As shown in Figure 7b, the luminescence quantum efficiency of monolayer MoS_2_ was much higher than that of multilayer and bulk MoS_2_. The reason the PL intensity increases with decreasing MoS_2_ layer number is that the decrease of $K_{relax}$ and the luminescence resonance state can match the direct exciton transfer. The PL phenomenon of monolayer MoS_2_ is an inherent property which is not caused by external disturbances.

### 3.7. Characterization of Monolayer MoS_2_ on SiO_2_/Si Substrate Under the 633 nm Laser Wavelength

**Figure 8:**
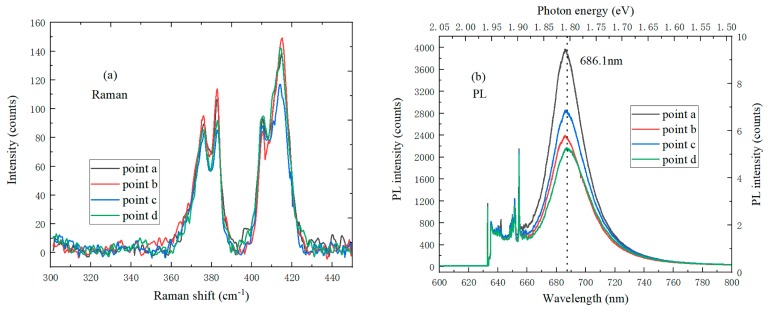
Optical properties of monolayer MoS_2_ on a SiO_2_/Si substrate under the laser wavelength of 633 nm (**a**) Raman spectrum; (**b**) PL spectrum.

The Raman spectrum of monolayer MoS_2_ on the SiO_2_/Si substrate was also tested under 633 nm laser wavelength, and the spectrum curve was fitted using the Gauss–Lorentz function, as shown in Figure 8a. The peak difference of monolayer MoS_2_ between the characteristic E^1^_2g_ and A_1g_ peaks was 20 cm^−1^, which preliminarily established that MoS_2_ was monolayer. It can be seen from Figure 8b that the PL spectrum has its strongest peak intensity at 686.1 nm, and the corresponding photon energy is 1.81 eV, which is consistent with the absorption transition peak of I excitation. Compared to the PL spectrum of monolayer MoS_2_ at 532 nm, it can be found that the position of the I exciton peak has a blueshift of 16 meV, and the PL intensity of monolayer MoS_2_ increases when the laser wavelength transforms from 532 to 633 nm. The reason is due to the transition of the upper energy level of spin cleavage valence band to the conduction band.

## 4. Conclusions

In this paper, large size and high-quality MoS_2_ layers were grown on the SiO_2_/Si and sapphire substrates by APCVD. The optical properties of MoS_2_ on the SiO_2_/Si and sapphire substrates were researched systematically by atomic force microscopy, optical microscopy, and Raman and photoluminescence spectroscopy under different laser light wavelengths and powers. The peak shift between characteristic A_1g_ and E^1^_2g_ peaks increased monotonously, and the I peak of the PL spectrum on the SiO_2_/Si substrate redshifted slightly when the layer number of MoS_2_ was increased, which can help to obtain the layer number and peak position of MoS_2_. In addition, the effects of layer number and external substrate environment on the optical properties of MoS_2_ were also studied systematically. The difference from monolayer MoS_2_ on the SiO_2_/Si substrate is that the B peak of the PL spectrum had a blueshift of 56 meV and the characteristic E^1^_2g_ peak of the Raman spectrum had no blueshift. The 1- and 2-layer MoS_2_ on the sapphire substrate has a higher PL peak intensity than that of the SiO_2_/Si substrate. When the laser wavelength transforms from 532 to 633 nm, the position of the I exciton peak had a blueshift of 16 meV, and the PL intensity of monolayer MoS_2_ on a SiO_2_/Si substrate increased. The optical properties of MoS_2_ can be obtained, which can pave the way for the fabrication of optoelectronic devices.
